# Supplementary material for: HIPK4 is a novel gene associated with teratozoospermia and male infertility
Source: Hum Reprod Open. 2026 Jul 20;2026(3):hoag066. doi: 10.1093/hropen/hoag066 (PMC13470646; doi:10.1093/hropen/hoag066)
Supplement: Supplementary file 1 [file hoag066_supplementary_data.zip › Manuscript HIPK4 supplementary data 2026-07-16.pdf]

Supplementary data

***HIPK4* is a novel gene associated with teratozoospermia and male infertility**

Sophie Adina Koser[1,2], Cynthia Rieck[3], Isabella Aprea[3], Claudia Krallmann[4], Avinash Satish Gaikwad[1], Julia Wallmeier[2], Retno Tenardi-Wenge[3], Sara Di Persio[5,6], Nina Neuhaus[5], Johanna Raidt[3], Heymut Omran[3], Sandra Laurentino[1], Sabine Kliesch[4], Birgit Stallmeyer[1], Corinna Friedrich[1,#], Frank Tüttelmann[1,2*,#]

[1]Institute of Reproductive Genetics (IRG), Centre of Medical Genetics (CMG), University of Münster, Münster, Germany

[2]Department of Medical Genetics, Centre of Medical Genetics (CMG), University Hospital Münster, Münster, Germany

[3]Department of General Paediatrics, University Hospital Münster, Münster, Germany

[4]Department of Andrology, Centre of Reproductive Medicine and Andrology (CeRA), University Hospital Münster, Münster, Germany

[5]Centre of Reproductive Medicine and Andrology (CeRA), University and University Hospital Münster, Münster, Germany

[6]Department of Anatomy, Histology, Forensic Medicine and Orthopedics, Section of Histology, Sapienza University of Rome, Rome, Italy

Table of Content

Supplementary Tables

Supplementary Figures

Supplementary Tables

Supplementary Table S1. Primer sequences.

Purpose	Primer sequence (5‘-3‘)
**Sanger validation of variants**	
HIPK4 c.1A>G	Forward: TCCAGCAGCTCAAAGACCAG; Reverse: TCACTCCTGCCTTATCCCCA
DNAH17 c.1076_1077dup (exon 8)	Forward: TAGGGTAGGAGCAGTCTGGG; Reverse: ATTTATGCTGCAGCCTTGGC
DNAH17 c.5932G>A (exon 39)	Forward: ATGAATACGGGCAGGTCGTC; Reverse: AGGAGGGAAGTAGGGAGCAG
DNAH17 c.7752+2T>A (exon 49)	Forward: CTGGCTAGCTGCTCATCCTC; Reverse: GCAGGAGAATGGGGCGAA
**Cloning and mutagenesis**	
Cloning of *HIPK4* 5’ UTR and exon 1 (complementary sequence in bold) introducing restriction enzyme sites and a premature stop codon (italic)	Forward (HindIII): GGTGGTAAGCTT**ACTCAACAGCGCTGGAACCCATTCGG**<br>Reverse (XhoI): ACCACCCTCGAG*TTA***CTTGACCCTGAAGGGGCAGCGGGTCT**
Subsequent cloning of HA-Tag using phosphorylated primers	Forward (HA-tag): TACCCATACGATGTTCCAGATTACGCTTAACTCGAGTCTAGAGGGCCCGTTTAAACC<br>Reverse: CTTGACCCTGAAGGGGCAGCGGGT
PCR and sequencing of the insert	Forward: GTAACAACTCCGCCCCATTG<br>Reverse: AGGAAAGGACAGTGGGAGTG
Cloning of HIPK4 5’ UTR and complete cDNA	Forward (HindIII): GGTGGTAAGCTT**ACTCAACAGCGCTGGAACCCATTCGG**

	Reverse (<u>XhoI</u>): ACCACC<u>CTCGAG</u>**TCAGTGGTGCCCGGTGACATGCT**
Subsequent cloning of HA-Tag using phosphorylated primers	Forward (<u>HA-tag</u>): <u>TACCCATACGATGTTCCAGATTACGCT</u>TGACTCGAGTCTAGAGGGCCCGTTTAAACC<br>Reverse: CTCCAGCATGTCACCGGGCACCAC
Mutagenesis c.1A>G	Forward: ACCGTGTCCACCATCCAGTCGGAGACTGA<br>Reverse: TCAGTCTCCGACTGGATGGTGGACACGGT
Sequencing of the HIPK4 construct	Insert primer 1: GTAACAACTCCGCCCCATTG<br>Insert primer 2: CTTCAGCAACCTCATTCGGC<br>Insert primer 3: AGGAAAGGACAGTGGGAGTG<br>Standard pcDNA3.1(+) primer:<br>AmpStart: AAAACAGGAAGGCAAAATGC<br>AmpStop: TCAGGCAACTATGGATGAAC<br>BGH Reverse: TAGAAGGCACAGTCGAGG<br>CMV Forward: CGCAAATGGGCGGTAGGCGTG<br>pCI Reverse: GCAATAGCATCACAAATTTCAC

Abbreviations: UTR: Untranslated region

Supplementary Table S2. Antibody information.

Antibody	Manufacturer	Identifier/Catalogue number	Dilution
**Immunofluorescence staining**			
**Primary antibody**			
Mouse anti-acetylated alpha-tubulin	Sigma-Aldrich by Merck, Darmstadt, Germany	T6793	1:4,000 in 1% BSA in PBS
**Secondary antibody**			
Goat anti-mouse Alexa Fluor 488®	Thermo Fischer Scientific, Waltham, MA, USA	A11029	1:1,000 1% BSA in PBS
**Western Blot**			
**Primary antibody**			
Rat anti-HA-tag	Roche by Merck, Darmstadt, Germany	11867423	1:2,000 in 5% milk powder in TBST
Rabbit anti-GAPDH	Cell Signaling Technology, Danvers, MA, USA	5174	1:1,500 in 5% milk powder in TBST
**Secondary antibody**			
Goat anti-rat	Sigma-Aldrich by Merck, Darmstadt, Germany	A9037	1:1,000 in 5% milk powder in TBST

Mouse anti-rabbit	Santa Cruz Biotechnology, Dallas, TX, USA	2357	1:1,000 in 5% milk powder in TBST

Abbreviations: BSA: Bovine serum albumin; PBS: Phosphate Buffered Saline; TBST: Tris-buffered saline with Tween20

Supplementary Table S3. Amino acid sequences and functional annotation.

Amino acid sequences (UniProt)

**Protein**	**Species**	**UniProt Identifier**
**HIPK4**	Human	Q8NE63
	Macaque	Q8WP28
	Mouse	Q3V016
	Rat	Q4V793
	Bovine	E1BB49
	Pig	I3LVJ2
	Horse	F6YYR8
**DNAH17**	Human	Q9UFH2
	Macaque	A0A2K6DCQ0
	Mouse	Q69Z23
	Rat	D4A599
	Bovine	E1BLB4
	Pig	A0A8W4FH26
	Horse	F7BXB8

	Platypus	F6SCH9
**Functional annotation**		
**Protein (UniProt ID)**	**Domain or binding site**	**Amino acids**
HIPK4 (UniProt: Q8NE63)	Protein kinase domain	11-347
	ATP binding site	17-25 and 40
	Active site	136
DNAH17 (UniProt: Q9UFH2)	ATPases associated with diverse cellular activities (AAA) domain 1	1809-2030
	ATP binding site	1847-1854

Abbreviations: ATP: Adenosine triphosphate

Supplementary Table S4. Rules for assignment of specific ACMG criteria.

ACMG criterion	Description
**PVS1**	PVS1 was assigned for variants leading to a premature stop codon (PMC) such as nonsense and frameshift variants that are expected to cause nonsense mediated decay (PMC > 50–55 nucleotides from the last exon-intron-junction) if loss-of function (LoF) is a known mechanism or for variants disrupting a canonical splice site (+/-1,2) likely leading to LoF. LoF was assumed for *HIPK4* based on published mouse models (Crapster *et al*., 2020; Liu *et al*., 2022).
**PS3**	Functional evidence supporting a deleterious effect led to the assignment of PS3 with one point per experiment (*in vitro* analysis of translation initiation and assessment of protein stability counted as one piece of evidence: PS3_supporting).
**PM2**	PM2 was given for variants that are absent in gnomAD (v4.1.0) and downgraded to supporting for variants with MAF < 1% in gnomAD (v4.1.0).
**PM3**	PM3 was assigned for variants *in trans* with a (likely) pathogenic variant in the same gene and downgraded to supporting in case of homozygosity or a potential alternative genetic cause that might explain the patient's condition instead.
**PM4**	Variants leading to an in-frame deletion or insertion on protein level received PM4.
**PP1**	PP1 was not assigned in this study due to identification of two different genetic causes rather than one shared among all affected individuals.
**PP3**	PP3 was assigned for missense variants with a CADD ≥20.

Based on the strength of evidence the corresponding points per criterion were added (very strong: 8; strong: 4; moderate: 2; supporting; 1) resulting in classification as pathogenic (≥ 10), likely pathogenic (6-9) or variants of uncertain significance (VUS, 0-5) (Tavtigian *et al*., 2020).

Supplementary Table S5. Explorative analysis of variants shared by the affected brothers.

Gene (transcript)	Exon	Variant	CADD	MAF gnomAD v2.1.1	Expression (GTEx)	Comments
**Homozygous variants shared by all four affected brothers**						
none						
**Homozygous variants shared by M1344, M1670, and M1611**						
PRODH2 (NM_021232.1)	11/11	c.1522G>T p.(Val508Leu)	24.4	0.000184	Not in the testis	Excluded
MAP4K1 (NM_001042600.3)	18/31	c.1214C>T p.(Ser405Phe)	26.5	0.00003393	Not in the testis	Excluded
HIPK4 (NM_144685.5)	1/4	c.1A>G p.(Met1?) changed to p.(Met1_Glu35del)	20.5	-	Highest in testis, also in the brain	Published mouse models with male infertility and oligoasthenoteratozoospermia (PMID: 32163033; PMID: 35931115)
**Compound-heterozygous variants shared by all four affected brothers**						
none						
**Compound-heterozygous variants shared by M1344, M1670, and M1611**						
none						
**Heterozygous variants (MAF gnomAD < 0.001) shared by all four affected brothers**						
none						
**Heterozygous variants (MAF gnomAD < 0.001) shared by M1344, M1670, and M1611**						

EFL1 (NM_024580.6)	16/20	c.1849A>C p.(Ile617Leu)	23.8	0.00001203	Ubiquitous	Autosomal recessive Shwachman-Diamond syndrome 2 (OMIM 617941); excluded
PHOSPHO1 (NM_001143804.1)	3/3	c.49_52dup p.(Leu18ArgfsTer353)	24.2	-	Testis and blood	*Phospho1*$^{-/-}$ mice have a skeletal phenotype, mating of *Phospho1*$^{+/-}$ mice (and of *Phospho1*$^{-/-}$ mice) produced pubs (PMID: 20684022); no entry in OMIM; no described association with human male infertility; excluded
**Hemizygous variants (MAF gnomAD < 0.001) shared by all four affected brothers**						
none						
**Hemizygous variants (MAF gnomAD < 0.001) shared by M1344, M1670, and M1611**						
none						

See Supplementary Fig. S2 for a detailed description of the workflow. Abbreviations: MAF: Minor allele frequency

Supplementary Table S6. Preliminary ClinGen curation for *HIPK4* as autosomal recessive gene associated with male infertility due to teratozoospermia with or without oligo- or asthenozoospermia.

Evidence category	Evidence type	Suggested points		Points given	PMID/Comments	Total
		Default	Range			
Experimental evidence (max. 6 points)						4
Function	Protein Interaction	0.5	0-2	0.5	Interaction with and phosphorylation of RIM-BP3 required for male fertility in mice (Liu et al., 2022; PMID: 35931115)	
	Expression	0.5	0-2	1	Expression almost limited to testis (and brain) (Uhlén et al., 2015; PMID: 25613900), single cell expression in spermatids (this study)	
Functional alteration	Non-patient cells	0.5	0-1	0.5	Cell shape changes, loss of stress fibres and cytokinesis failure after expression in somatic cells (Crapster et al., 2020; PMID: 32163033)	
Models	Non-human model organism	2	0-4	2	Two independent infertile mouse models with OAT (Crapster et al., 2020; PMID: 32163033; Liu et al., 2022; PMID: 35931115)	
Genetic evidence (max. 12 points)						3
Variant evidence	Predicted or proven null variant (1.5 points)	0.5 (per variant)	0 - 3 (per variant)	3	c.1A>G p.(Met1_Glu35del), homozygous, this study	
	Other variant type (0.1 points)	0.1 points (per variant)	0 - 1.5 points (per variant)	0	c.935C>T p.(Ala312Val), homozygous, not counted due to a different phenotype (azoospermia and maturation arrest) and lack of functional validation (Alhathal et al., 2020; PMID: 32719396)	
Combined experimental and genetic evidence						**7***
				LIMITED	0.1-6	
				**MODERATE**	**7-11**	
				STRONG	12-18	
				DEFINITIVE	12-18<br>+ Replicated over time	

*Due to limited genetic evidence it is impossible to establish the gene-disease relationship despite the preliminary score of 7 points. Identification of at least two more unrelated patients with (likely) pathogenic *HIPK4* variants in homozygous or compound heterozygous state and a similar phenotype is mandatory to introduce *HIPK4* as a diagnostic gene.

Abbreviations: OAT: Oligoasthenoteratozoospermia.

Supplementary Table S7. ACMG classification of variants.

Gene (transcript): variant	ACMG criteria	ACMG class
**This study**		
DNAH17 (NM_173628.4): c.1076_1077dup p.(Lys360*)	PVS1 (LoF), PM2_supporting (MAF 0.00003603), PM3 (*in trans* with c.7752+2T>A p.? in M865)	pathogenic
DNAH17 (NM_173628.4): c.7752+2T>A p.?	PVS1, PM2_supporting (MAF 0.0003591), PM3 (*in trans* with c.1076_1077dup p.(Lys360*) in M865)	pathogenic
DNAH17 (NM_173628.4): c.5932G>A p.(Glu1978Lys)	PM2_supporting (MAF 0.00002360), PM3_supporting (*in trans* with c.7752+2T>A p.? in M1344 and M1611 but alternative genetic cause identified in both patients), PP3 (CADD GRCh37-v1.6: 27.6)	VUS
HIPK4 (NM_144685.5): c.1A>G p.(Met1_Glu35del)	PS3_supporting (*in vitro* assays showing truncation and reduced stability), PM2 (absent from gnomAD), PM3_supporting (homozygous), PM4 (inframe deletion N-terminal)	likely pathogenic
**Alhathal *et al.*, 2020**		
HIPK4 (NM_144685.3): c.935C>T p.(Ala312Val)	PM2_supporting (MAF 0.00001302), PM3_supporting (homozygous), PP3 (CADD GRCh37-v1.6: 22.7)	VUS

Abbreviations: ACMG: American College of Medical Genetics and Genomics; MAF: minor allele frequency (gnomAD, v4); VUS: variant of uncertain significance

Supplementary Figures

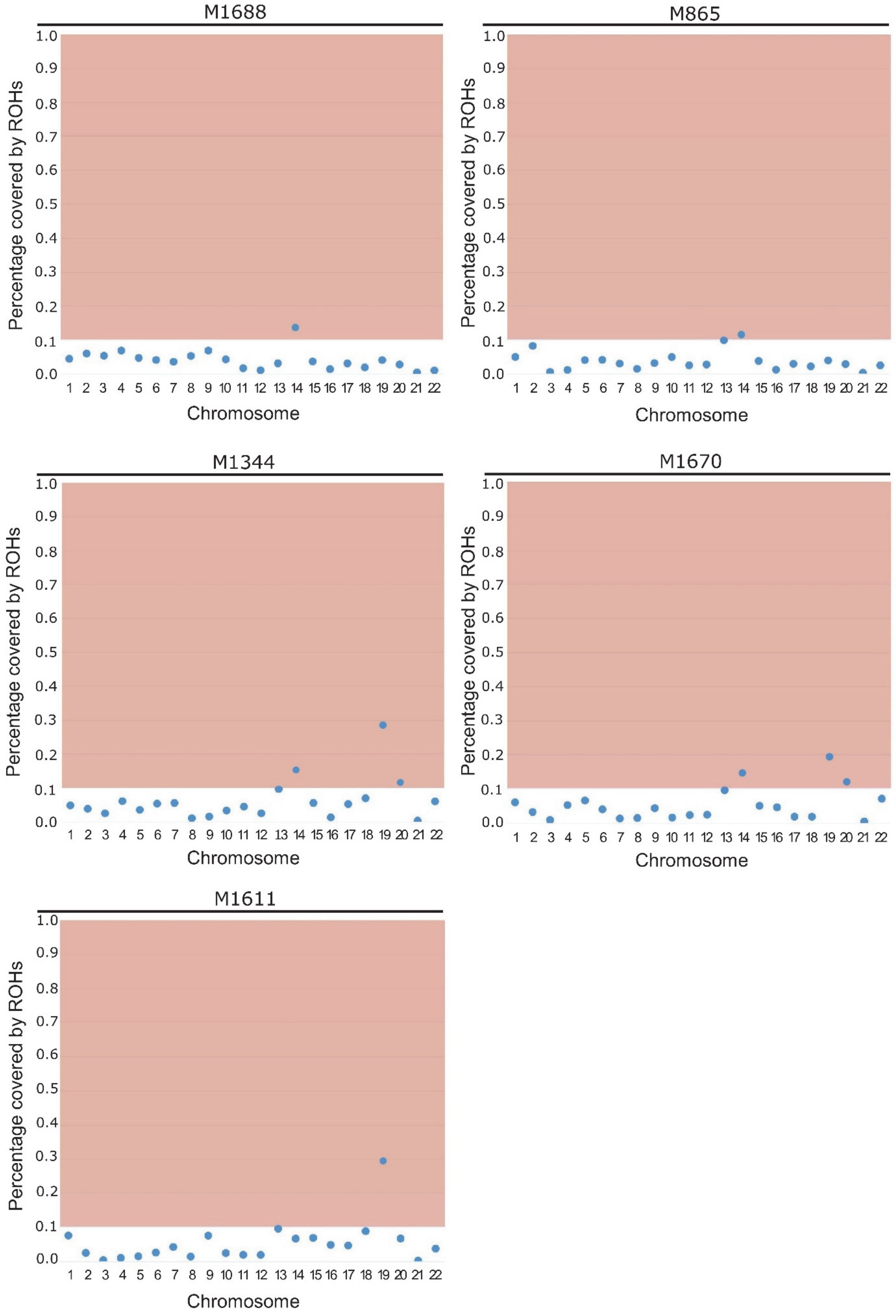

Supplementary Figure S1. Exome sequencing data shows that the parents are distantly related.

The exomes of mother, father, and their sons were analysed for regions of homozygosity (ROHs) using the AltAF plotter (Radtke *et al*., 2024). Using the cut-off proposed by the authors (≥ 3 chromosomes with ≥ 0.1 ROH) led to inconclusive results. Exome data of M1344 and M1670 comprises ROH above the threshold. M1344, M1670 and M1611 have increased runs of homozygosity in chromosome 19 including *HIPK4*. The data is compatible with a shared, distant ancestry of the parents.

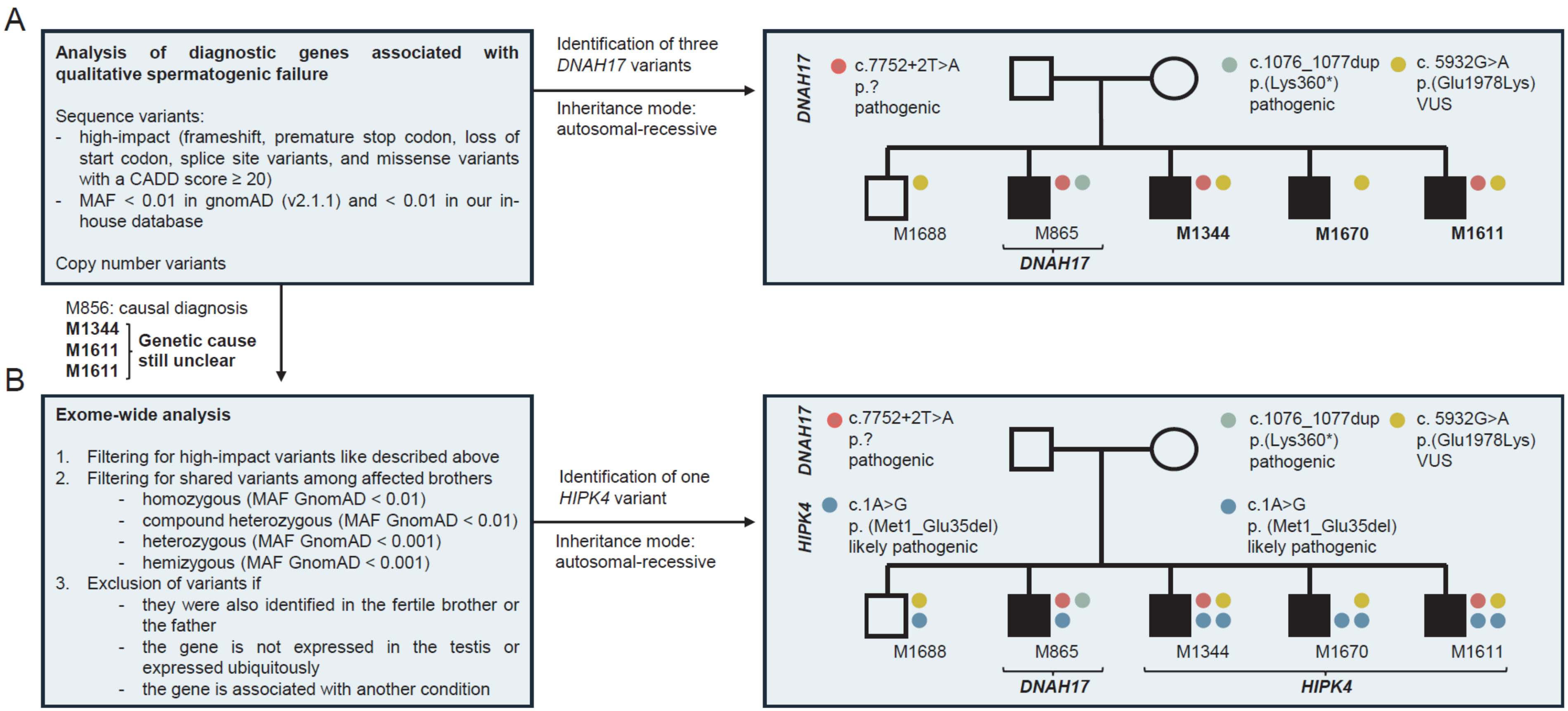

Supplementary Figure S2. Workflow and main results of the exome-based family analysis.

(A) 32 genes with an at least moderate level of evidence supporting their gene disease relationship with male infertility and astheno- or teratozoospermia (including other or mixed phenotypes) according to Stallmeyer et al. (2025) were included in the analysis: *ACTL7A, ARMC2, AURKC, CFAP43, CFAP44, CFAP58, CFAP61, CFAP65, CFAP69, CFAP70, CFAP91, CFAP251, DNAH1, DNAH2, DNAH6, DNAH8, DNAH10, DNAH17, DNHD1, DPY19L2, DRC1, FANCM, FSIP2, PLCZ1, PMFBP1, QRICH2, SPAG6, SPEF2, SSX1, SUN5, TSGA10, TTC29*. All analysed

genes follow an autosomal recessive (AR) inheritance pattern except for *SSX1* (X-linked). Except for the depicted variants in *DNAH17* (NM_173628.4), no biallelic variants nor hemizygous variants were identified in the affected brothers. Copy number variant (CNV) analysis did not identify any CNV in the analysed genes.

(B) The results of the exome-wide analysis of shared variants are shown in Supplementary Table S6. The homozygous variant in *HIPK4* (NM_144685.5) was identified as the most plausible cause of infertility in M1344, M1670, and M1611.

Abbreviations: MAF: minor allele frequency.

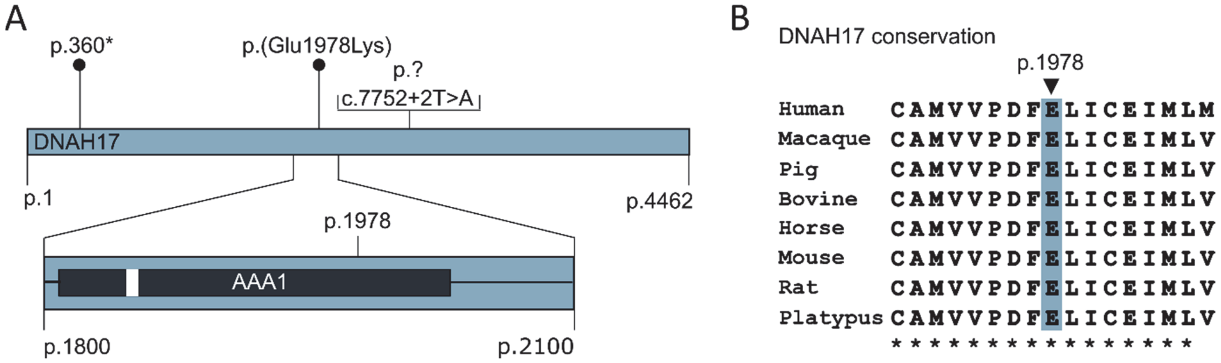

Supplementary Figure S3. Identified DNAH17 variants on protein level.

(A) *DNAH17* (NM_173628.4) codes for a protein with 4462 amino acids. The location of identified DNAH17 variants is depicted. Note that c.7752+2T>A disrupts the canonical splice site after exon 49 of 81 and is regarded as loss-of-function variant through aberrant splicing although the exact consequence at protein level is unknown (p.?). The missense variant p.(Glu1978Lys) is located within the first of six ‘ATPases associated with diverse cellular activities’ (AAA) domains (UniProt: Q9UFH2). The ATP binding site within this domain is marked in white. (B) DNAH17 amino acid sequences from different species were retrieved from UniProt. The Glutamic acid at position 1978 is conserved among mammals.

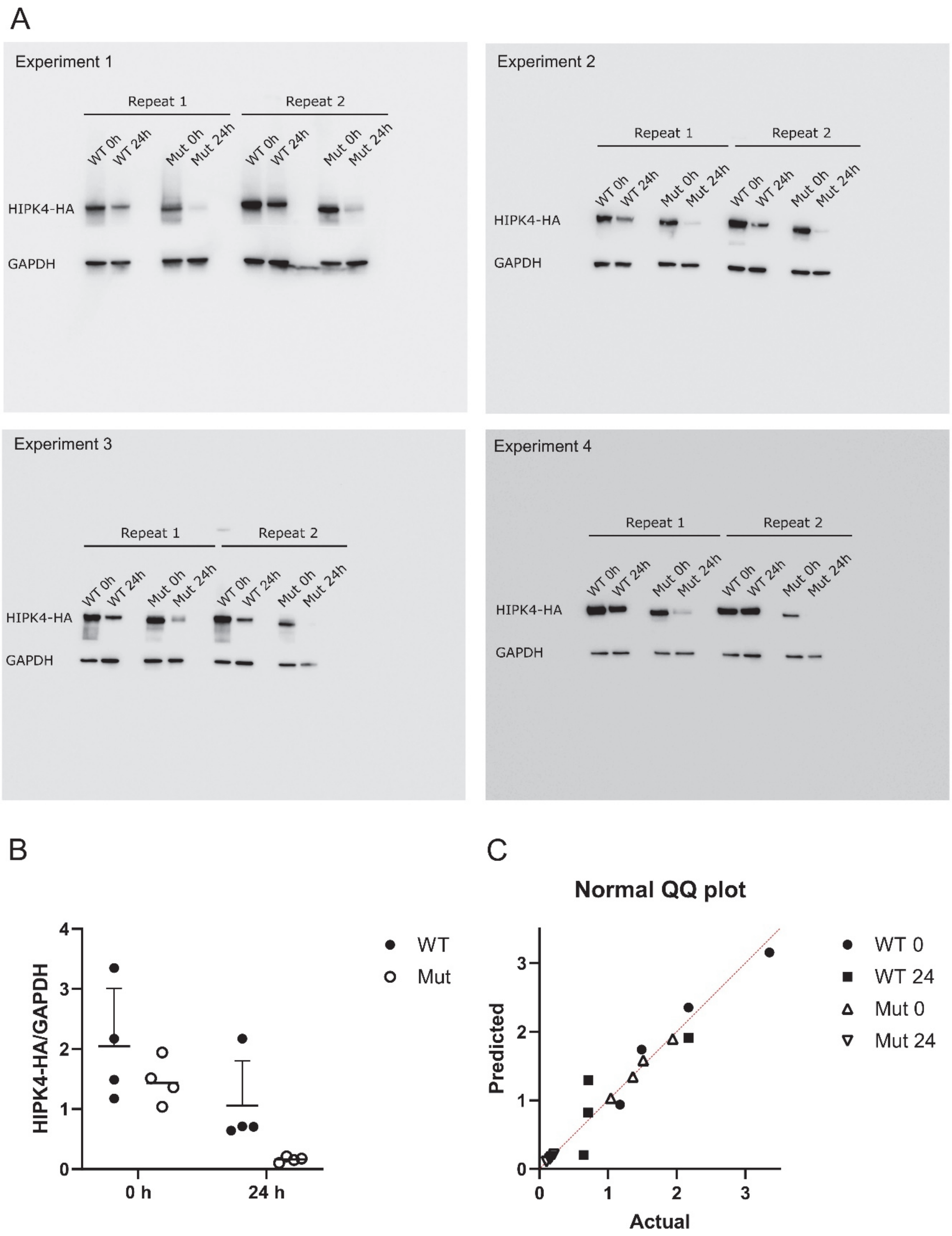

Supplementary Figure S4. Extended data of the cycloheximide assay.

(A) The experiment was carried out n = 4 times independently with two technical replicates that were analysed within the same Western blot with imaging of HIPK4-HA and GAPDH simultaneously. Quantification was performed by measuring mean grey values within a rectangular selection used throughout the blot. The background was measured directly above or below the respective band and subtracted. (B) Technical replicates were averaged and

plotted with mean and standard deviation. (C) Visual inspection of the QQ plot was done in advance of the statistical analysis.
